# Maternal Prepregnancy Body Mass Index and Gestational Weight Gain on Pregnancy Outcomes

**DOI:** 10.1371/journal.pone.0082310

**Published:** 2013-12-20

**Authors:** Nan Li, Enqing Liu, Jia Guo, Lei Pan, Baojuan Li, Ping Wang, Jin Liu, Yue Wang, Gongshu Liu, Andrea A. Baccarelli, Lifang Hou, Gang Hu

**Affiliations:** 1 Tianjin Women's and Children's Health Center, Tianjin, China; 2 Chronic Disease Epidemiology Laboratory, Pennington Biomedical Research Center, Baton Rouge, Louisiana, United States of America; 3 Departments of Epidemiology and Environmental Health, Harvard School of Public Health, Boston, Massachusetts, United States of America; 4 Department of Preventive Medicine, Feinberg School of Medicine, Northwestern University, Chicago, Illinois, United States of America; Erasmus Medical Center, Netherlands

## Abstract

**Objective:**

The aim of the present study was to evaluate the single and joint associations of maternal prepregnancy body mass index (BMI) and gestational weight gain (GWG) with pregnancy outcomes in Tianjin, China.

**Methods:**

Between June 2009 and May 2011, health care records of 33,973 pregnant women were collected and their children were measured for birth weight and birth length. The independent and joint associations of prepregnancy BMI and GWG based on the Institute of Medicine (IOM) guidelines with the risks of pregnancy and neonatal outcomes were examined by using Logistic Regression.

**Results:**

After adjustment for all confounding factors, maternal prepregnancy BMI was positively associated with risks of gestational diabetes mellitus (GDM), pregnancy-induced hypertension, caesarean delivery, preterm delivery, large-for-gestational age infant (LGA), and macrosomia, and inversely associated with risks of small-for-gestational age infant (SGA) and low birth weight. Maternal excessive GWG was associated with increased risks of pregnancy-induced hypertension, caesarean delivery, LGA, and macrosomia, and decreased risks of preterm delivery, SGA, and low birth weight. Maternal inadequate GWG was associated with increased risks of preterm delivery and SGA, and decreased risks of LGA and macrosomia, compared with maternal adequate GWG. Women with both prepregnancy obesity and excessive GWG had 2.2–5.9 folds higher risks of GDM, pregnancy-induced hypertension, caesarean delivery, LGA, and macrosomia compared with women with normal prepregnancy BMI and adequate GWG.

**Conclusions:**

Maternal prepregnancy obesity and excessive GWG were associated with greater risks of pregnancy-induced hypertension, caesarean delivery, and greater infant size at birth. Health care providers should inform women to start the pregnancy with a BMI in the normal weight category and limit their GWG to the range specified for their prepregnancy BMI.

## Introduction

Improvements of maternal, fetal, and child health are key public health goals. In recent years, maternal prepregnancy body mass index (BMI) has increased among the childbearing age women in developed countries [1]. It has been shown that women who are overweight or obese at the start of pregnancy are at increased risks of poor maternal and child health outcomes. Several recent studies reported that prepregnancy BMI was positively associated with infant birth weight [2], [3]. Furthermore, women who gain weight excessively or inadequately during pregnancy are at increased risks of poor maternal and child health outcomes [4]–[6]. Weight gain during pregnancy within the recommended range (11 to 40 pounds) remained constant during the last 10 years [7]. Several studies have shown that maternal excessive gestational weight gain (GWG) was associated with increased risks of pregnancy-induced hypertension, gestational diabetes mellitus (GDM), caesarean delivery and large for gestational age infant, and maternal inadequate GWG was associated with increased risks of low birth weight and small for gestational age infant [4]–[6]. The Danish National Birth Cohort found that excessive GWG increased risks of caesarean delivery and large for gestational age infant, and inadequate GWG increased the risk of having a small baby [3].

In 2009, the Institute of Medicine (IOM) published new recommendations for weight gain during pregnancy [8]. A recent US study reported that 73% of pregnant women had excessive GWG according to 2009 IOM guidelines [9]. The IOM guidelines based on different prepregnancy BMI are not only suitable for women in developed countries, but also suitable for Chinese women [10]. It has been shown that being prepregnancy overweight or obese and having an excessive GWG, as well as being underweight and having an inadequate GWG, were associated with increased risks for adverse pregnancy outcomes in women from China and other countries as well [11]. However, few studies estimated the joint associations of maternal prepregnancy BMI and GWG with pregnancy outcomes [3], [9]. Therefore, the aim of the present study was to evaluate the single and joint associations of maternal prepregnancy BMI and GWG with pregnancy outcomes in Tianjin, China.

## Methods

### Study Sample

Tianjin is the fourth largest city with over 12.9 million residents in northern China, and 4.3 million residents live in six central urban districts. Tianjin consists of 16 county-level administrative areas, including six central urban districts, one new urban district, and nine counties that govern towns and rural areas. The prenatal care and children health care in six central urban districts are a routine of a three-tier care system consisting of approximately 65 primary hospitals, 6 district-level Women's and Children's Health Centers (also including secondary hospitals), and a city-level (Tianjin) Women's and Children's Health Center (also including tertiary hospitals). In Tianjin, all pregnant women are registered at the primary hospitals, and in the 32^nd^ gestational week, they are referred to a secondary hospital or a tertiary hospital for management till delivery. All children are given health examinations in the postnatal period, infancy, and at preschool. Tianjin Women and Children's Health Center is the leader of the 3-tier care system and responsible for organization, co-ordination and implementation of women and child health care, research and promotion projects.

Health care records for both pregnant women and their children have been collected and available in electronic form since 2009 [12], [13]. Pregnant Women Health Records start within the first 12 weeks of pregnancy, and include general information (age, occupation, education, date of first visit, numbers of pregnancy/infants, last menstrual period, expected delivery date, smoking habits, etc), history of diseases, family history of diseases, clinical measurements (height, weight, blood pressure, gynaecological examinations, ultrasonography, GDM screening test and other lab tests), complications during pregnancy, pregnancy outcomes (delivery modes, labor complications, etc), and postnatal period examinations (<42 days after delivery) [13]. Children Health Records include information from newborns (date of birth, sex, gestational weeks of birth, birth weight, birth recumbent length, Apgar score, etc), postnatal period (<42 days after birth) (names of the child and his/her parents, family history of diseases, feeding modalities, weight, and recumbent length) [13]. We collected 43,854 records of both mothers and their infants who were born in the central urban districts between June 2009 and May 2011. The present study included 33,973 mother-child pairs (77.5%) with all information and clinical measurements after excluding multiple births (n = 987), stillbirth (n = 143), multiparous women (n = 2), and mother-child pairs missing any variables required for this analysis (n = 8,749). Compared with mothers excluded in the present study, the mothers included were younger (27.6 vs. 27.8 years old) and had a lower prepregnancy BMI (22.1 vs. 22.6 kg/m^2^). The study and analysis plan was approved by the Tianjin Women's and Children's Health Center Institutional Review Board. Tianjin Women's and Children's Health Center has agreed to waive the need for written informed consent from all participants involved in our study because we use the electronic dataset from health care records.

### Measurements

Mothers' anthropometric data were collected during the pregnancy by specially trained gynecologists in the primary hospitals by using the same devices. Weight and height were measured in light clothing and no shoes using a beam balance scale (RGZ-120, Jiangsu Suhong Medical Instruments Co., China). Blood pressure was measured using a standardized mercury sphygmomanometer (XJ11D, Shanghai Medical Instruments Co., China). Weight was measured to the nearest 0.01 kg using a digital scale (TCS-60, Tianjin Weighing Apparatus Co., China). Length was measured to the nearest 0.1 cm using a recumbent length stadiometer (YSC-2, Beijing Guowangxingda, China). We have done a validity study to compare the electronic data of measurements of birth weight and hospitals' measurements of birth weight among 454 children in six major hospitals. The correlation between two measurements is 0.991. We have also done a validity study to compare the electronic data of measurements of height and weight with the same visit's measurements of height and weight by trained health workers among 200 pregnancy women in four different local health centers. The correlations between electronic data and measurement data are 0.998 for body weight and 0.997 for height in these pregnancy women.

Body mass index (BMI) was calculated by dividing weight in kilograms by the square of height in meters. Prepregnancy BMI was categorized as underweight (BMI<18.5 kg/m^2^), normal-weight (18.5 kg/m^2^≤BMI<24 kg/m^2^), overweight (24 kg/m^2^≤BMI<28 kg/m^2^), or obese (BMI≥28 kg/m^2^) using the standard of Working Group on Obesity in China[14]. The Chinese BMI classification standard was used due to the best sensitivity and specificity for identifying risk factors including hypertension, type 2 diabetes, and dyslipidemia in the Chinese population [15]–[17]. The prepregnancy BMI was calculated using the weight and height recorded at the first prenatal visit within the first 12 weeks of pregnancy. A previous study reported that there was a high correlation between self-reported prepregnancy weight and weight recorded at the first visit [18]. Weight gain of mothers during pregnancy was calculated as the difference between prepregnancy and delivery weight. Adequacy of GWG was defined according to the Chinese maternal prepregnancy BMI status and the 2009 IOM GWG recommendations (1): 12.5–18 kg (prepregnancy BMI<18.5 kg/m^2^), 11.5–16 kg (BMI 18.5–23.9 kg/m^2^), 7–11.5 kg (BMI 24.0–27.9 kg/m^2^), and 5–9 kg (BMI>28 kg/m^2^) [8]. We used the translation of US IOM GWG recommendations because no official recommendations exist in China.

We considered the risks of GDM, pregnancy-induced hypertension, caesarean section, preterm birth (preterm delivery), large for gestational age infant, small for gestational age infant, macrosomia and low birth weight as pregnancy complications and pregnancy outcomes. GDM was diagnosed based on a 75-g 2-hour oral glucose tolerance test (OGTT) at pregnancy 24–28 weeks [19]. Women with impaired glucose tolerance (IGT) (fasting glucose <126 mg/dL and 2-hour glucose ≥140 and <200 mg/dL) and diabetes (fasting glucose ≥126 mg/dL or 2-hour glucose ≥200 mg/dL) were defined as GDM according to WHO diagnostic criteria [20]. Pregnancy-induced hypertension was diagnosed by a systolic blood pressure ≥140 mmHg or diastolic blood pressure ≥90 mmHg in the 3^rd^ trimester or using antihypertensive drugs [21]. Preterm delivery was defined as gestational weeks of delivery <37 weeks. Z scores for birth weight for gestational age, and birth length for gestational age were calculated using our own study population means and standard deviations. A small-for-gestational-age (SGA) infant was defined as an infant having a standardized birth weight <10th percentile, whereas a large-for-gestational-age (LGA) infant was defined as an infant having a standardized birth weight >90th percentile. Neonatal outcomes also included low birth weight (birth weight <2500 g) and macrosomia (birth weight ≥4000 g).

### Statistical analyses

The general characteristics of both mothers and children based on different categories of maternal prepregnancy BMI and GWG were compared using the General Linear Model and chi-square test. Logistic regression was used to assess the single and joint associations of maternal prepregnancy BMI and GWG with the risks of pregnancy and neonatal outcomes. The analyses were adjusted for maternal age, maternal height, maternal education, smoking, family income, maternal occupation, gestational age, and birth weight (if needed). The significance of the trend over different categories of maternal prepregnancy BMI and GWG categories was tested in the same models by giving an ordinal numeric value for each dummy variable. The criterion for statistical significance was <0.05 (for two-sided tests). All statistical analyses were performed with PASW for Windows, version 20.0 (Statistics 20, SPSS, IBM, USA)

## Results

The general characteristics of both mothers and children based on maternal prepregnancy BMI and GWG categories are presented in Table 1. Mothers who were overweight or obese before pregnancy were older, and had a lower education level and a lower family income compared with mothers with prepregnancy normal weight. Compared with mothers with adequate GWG, mothers with excessive GWG were younger, had a higher prepregnancy BMI, and reported a lower education level, and mothers with inadequate GWG reported a lower education level and a lower family income.

**Table 1:** Characteristics of study participants among 33-infant pairs according to maternal prepregnancy body mass index and gestational weight gain categories in Tianjin, China.

	Prepregnancy body mass index (kg/m^2^)				*P*	Institute of Medicine categories			*P*
	<18.5	18.5–23.9	24.0–27.9	≥28		Inadequate	Adequate	Excessive	
No. of subjects	3 809	21 942	6 185	2 037		3 340	11 227	19 406	
**Maternal characteristics**									
Gestational weight gain, kg	16.5 (5.1)	17.7 (5.5)	18.1 (6.2)	17.3 (6.8)	<0.001	9.4 (1.9)	14.3 (1.8)	21.0 (5.1)	<0.001
Maternal age before pregnancy, y	26.8 (2.9)	27.6 (3.1)	28.0 (3.4)	28.0 (3.4)	<0.001	27.7 (3.6)	27.8 (3.2)	27.5 (3.1)	<0.001
Prepregnancy body mass index, kg/m^2^	17.5 (0.8)	21.1 (1.5)	25.6 (1.1)	30.5 (2.4)	<0.001	20.5 (2.4)	20.9 (2.5)	23.1 (3.6)	<0.001
Gestational age at delivery, wk	39.1 (1.3)	39.2 (1.3)	39.1 (1.4)	38.9 (1.5)	<0.001	39.0 (1.5)	39.1 (1.4)	39.2 (1.3)	<0.001
Caesarean delivery, %	55.1	63.1	75.3	83.6	<0.001	57.6	59.3	70.7	<0.001
Blood pressure during third trimester, mmHg									
Systolic	105 (10.1)	108 (10.4)	112 (10.8)	116 (11.6)	<0.001	106 (10.4)	107 (10.4)	110 (10.8)	<0.001
Diastolic	67 (7.0)	69 (7.5)	72 (7.8)	75 (8.6)	<0.001	68 (7.6)	69 (7.6)	70 (7.8)	<0.001
Mother's education, %					<0.001				<0.001
University and above	44.7	48.3	41.6	30.5		42.9	49.3	44.0	
Junior college	28.8	26.9	27.9	27.3		25.7	26.2	28.2	
High school and under	26.5	24.8	30.5	42.2		31.4	24.5	27.8	
Family income, yuan/month, %					<0.001				<0.001
≥3000	55.7	58.1	51.8	43.8		50.5	57.7	55.7	
2000–2999	23.8	21.8	25.4	25.7		23.9	22.3	23.1	
<2000	20.5	20.1	22.8	30.5		25.6	20.0	21.2	
Occupation of mother, %					<0.001				<0.001
Industrial workers	15.5	16.3	19.5	23.8		17.6	16.2	17.8	
Office workers	42.7	43.3	39.7	34.4		40.6	43.0	41.7	
Service professional workers	19.3	21.2	19.5	16.6		18.9	21.6	19.9	
Unemployed persons	10.2	8.2	9.2	11.8		10.3	8.2	9.0	
Other	12.3	11.0	12.1	13.4		12.6	11.0	11.6	
Smoking during pregnancy, %	1.2	1.0	1.2	2.2	<0.001	0.9	0.8	1.4	<0.001
Passive smoking, %	48.2	48.5	50.1	54.0	<0.001	46.9	46.6	50.9	<0.001
**Child characteristics**									
Boy, %	50.0	51.9	52.0	52.6	0.131	53.1	51.0	52.0	0.062
Preterm delivery, %	2.6	2.9	3.4	4.9	<0.001	5.0	3.4	2.6	<0.001
Large for gestational age, %	4.0	9.1	14.9	22.6	<0.001	4.6	6.2	13.9	<0.001
Small for gestational age, %	15.5	9.0	6.9	5.7	<0.001	15.5	11.3	6.8	<0.001
Macrosomia (birth weight ≥4000 g), %	4.0	8.6	14.1	20.0	<0.001	4.2	5.8	13.0	<0.001
Low birth weight (<2500 g), %	2.9	2.0	2.2	2.9	0.001	3.8	2.7	1.7	<0.001

Large for gestational age was defined as birth weight >90^th^ percentile; Small for gestational age was defined as birth weight <10^th^ percentile.

Table 2 shows the relative risks of maternal outcomes by single and joint effects of maternal prepregnancy BMI and GWG. Numbers of subjects of maternal outcomes by joint effects of maternal prepregnancy BMI and weight gain during pregnancy are presented in Table S1. After adjustment for all confounding factors, maternal prepregnancy BMI was positively associated with risks of GDM, pregnancy-induced hypertension, caesarean delivery, and preterm delivery. Maternal excessive GWG was associated with increased risks of pregnancy-induced hypertension and caesarean delivery, and a decreased risk of preterm delivery, and maternal inadequate GWG was associated with an increased risk of preterm delivery, compared with maternal adequate GWG. In the joint analyses of maternal prepregnancy BMI and GWG with maternal outcomes, the positive associations of prepregnancy BMI with the risks of GDM, pregnancy-induced hypertension, caesarean delivery, and preterm delivery were consistent in subjects with different levels of GWG. Women with both prepregnancy obesity and excessive GWG or adequate GWG had the highest (2.2–7.1 folds) risks of GDM, pregnancy-induced hypertension, and caesarean delivery compared with women with normal prepregnancy BMI and adequate GWG.

**Table 2:** Odd ratios (95% confidence intervals) of maternal outcomes by joint effects of maternal prepregnancy body mass index and weight gain during pregnancy.

Prepregnancy body mass index (kg/m^2^)	Institute of Medicine categories			P for trend	Total
	Inadequate	Adequate	Excessive		
Gestational diabetes (n = 1721)*					
<18.5	0.51 (0.31–0.83)	0.65 (0.49–0.87)	0.35 (0.22–0.56)	0.056	0.59 (0.48–0.74)
18.5–23.9	1.29 (1.06–1.56)	1.00	0.72 (0.63–0.84)	<0.001	1.00
24.0–27.9	1.78 (0.85–3.75)	2.56 (2.00–3.28)	1.61 (1.39–1.85)	0.001	1.91 (1.70–2.14)
≥28.0	0.77 (0.10–5.75)	3.57 (2.03–6.28)	2.18 (1.81–2.63)	0.140	2.46 (2.09–2.90)
P for trend	0.002	<0.001	<0.001		<0.001
Total	1.05 (0.88–1.24)	1.00	1.02 (0.92–1.14)	0.857	
Pregnancy-induced hypertension (n = 742)*					
<18.5	0.39 (0.14–1.06)	0.46 (0.25–0.86)	0.75 (0.41–1.37)	0.375	0.45 (0.31–0.67)
18.5–23.9	1.13 (0.79–1.63)	1.00	1.35 (1.07–1.71)	0.039	1.00
24.0–27.9	0.78 (0.11–5.68)	2.55 (1.67–3.88)	2.41 (1.89–3.07)	0.521	2.03 (1.70–2.43)
≥28.0	6.61 (1.50–29.2)	7.08 (3.53–14.2)	5.94 (4.62–7.65)	0.833	5.07 (4.17–6.16)
P for trend	0.012	<0.001	<0.001		<0.001
Total	0.89 (0.65–1.24)	1.00	1.93 (1.62–2.31)	<0.001	
Caesarean section (n = 22 297)#					
<18.5	0.86 (0.74–1.01)	0.87 (0.78–0.96)	1.20 (1.06–1.36)	<0.001	0.83 (0.78–0.90)
18.5–23.9	0.99 (0.90–1.09)	1.00	1.34 (1.26–1.43)	<0.001	1.00
24.0–27.9	1.29 (0.81–2.04)	1.53 (1.29–1.81)	1.94 (1.79–2.10)	0.012	1.62 (1.52–1.73)
≥28.0	1.77 (0.69–4.56)	3.69 (2.07–6.58)	2.86 (2.51–3.26)	0.501	2.49 (2.20–2.81)
P for trend	0.108	<0.001	<0.001		<0.001
Total	0.96 (0.88–1.04)	1.00	1.54 (1.46–1.62)	<0.001	
Preterm delivery (n = 1050)§					
<18.5	1.34 (0.92–1.95)	0.74 (0.54–1.02)	0.55 (0.36–0.86)	0.005	0.93 (0.75–1.15)
18.5–23.9	1.51 (1.22–1.87)	1.00	0.61 (0.51–0.72)	<0.001	1.00
24.0–27.9	1.65 (0.66–4.10)	1.65 (1.18–2.31)	0.90 (0.74–1.09)	0.003	1.15 (0.98–1.35)
≥28.0	2.52 (0.59–10.77)	0.91 (0.29–2.88)	1.49 (1.18–1.90)	0.557	1.70 (1.36–2.11)
P for trend	0.786	0.006	<0.001		<0.001
Total	1.47 (1.22–1.77)	1.00	0.77 (0.67–0.88)	<0.001	

* Adjusted for maternal age, maternal height, maternal education, smoking, family income, maternal occupation, and gestational age.

# Adjusted for maternal age, maternal height, maternal education, smoking, family income, maternal occupation, gestational age, and birth weight.

§ Adjusted for maternal age, maternal height, maternal education, smoking, family income, and maternal occupation.

Table 3 shows that the relative risks of neonatal outcomes by single and joint effects of maternal prepregnancy BMI and GWG. Numbers of subjects of neonatal outcomes by joint effects of maternal prepregnancy BMI and weight gain during pregnancy are presented in Table S1. After adjustment for all confounding factors, maternal prepregnancy BMI was positively associated with risks of LGA and macrosomia, and inversely associated with risks of SGA and low birth weight. Maternal excessive GWG was associated with increased risks of infant LGA and macrosomia, and decreased risks of infant SGA and low birth weight, and maternal inadequate GWG was associated with an increased risk of infant SGA, and decreased risks of infant LGA and macrosomia at birth, compared with maternal adequate GWG. The positive associations of maternal prepregnancy BMI with the risks of infant LGA and macrosomia, and the inverse associations of maternal prepregnancy BMI with the risks of infant SGA and low birth weight were consistent in mothers with different levels of GWG except in obese mothers with inadequate and adequate GWG. Infants born to mothers with prepregnancy obesity and excessive GWG had the highest (4.0–4.1 folds) risk of LGA and macrosomia, infants born to mothers with both prepregnancy lean (BMI<18.5) and inadequate GWG had the highest (2.2 folds) risk of SGA, compared with those children born to mothers with both prepregnancy normal weight and adequate GWG.

**Table 3:** Odd ratios (95% confidence intervals) of neonatal outcomes by joint effects of maternal prepregnancy body mass index and weight gain during pregnancy.

Prepregnancy body mass index (kg/m^2^)	Institute of Medicine categories			P for trend	Total
	Inadequate	Adequate	Excessive		
Large for gestational age (n = 3 544)*					
<18.5	0.31 (0.18–0.51)	0.45 (0.34–0.60)	1.00 (0.79–1.27)	<0.001	0.42 (0.36–0.50)
18.5–23.9	0.77 (0.63–0.94)	1.00	1.91 (1.72–2.12)	<0.001	1.00
24.0–27.9	1.26 (0.58–2.75)	1.48 (1.14–1.93)	2.60 (2.32–2.92)	<0.001	1.73 (1.59–1.88)
≥28.0	3.81 (1.40–10.4)	3.32 (2.01–5.50)	3.99 (3.47–4.59)	0.774	2.80 (2.49–3.15)
P for trend	<0.001	<0.001	<0.001		<0.001
Total	0.72 (0.60–0.87)	1.00	2.32 (2.12–2.53)	<0.001	
Small for gestational age (n = 3 112)*					
<18.5	2.20 (1.81–2.67)	1.87 (1.62–2.15)	0.97 (0.79–1.19)	<0.001	1.84 (1.66–2.03)
18.5–23.9	1.45 (1.27–1.65)	1.00	0.68 (0.61–0.75)	<0.001	1.00
24.0–27.9	0.63 (0.27–1.44)	0.84 (0.65–1.09)	0.64 (0.56–0.73)	0.165	0.75 (0.67–0.83)
≥28.0	1.21 (0.36–4.09)	1.06 (0.55–2.05)	0.51 (0.41–0.63)	0.032	0.61 (0.50–0.74)
P for trend	0.001	<0.001	<0.001		<0.001
Total	1.41 (1.26–1.57)	1.00	0.60 (0.55–0.65)	<0.001	
Macrosomia (n = 3 318)#					
<18.5	0.32 (0.19–0.55)	0.49 (0.37–0.65)	1.04 (0.81–1.33)	<0.001	0.45 (0.38–0.53)
18.5–23.9	0.80 (0.65–0.99)	1.00	1.90 (1.71–2.12)	<0.001	1.00
24.0–27.9	0.96 (0.38–2.39)	1.69 (1.30–2.21)	2.65 (2.35–2.99)	0.001	1.76 (1.62–1.93)
≥28.0	3.08 (1.01–9.39)	3.49 (2.03–6.01)	4.10 (3.53–4.75)	0.676	2.86 (2.53–3.23)
P for trend	0.002	<0.001	<0.001		<0.001
Total	0.73 (0.60–0.88)	1.00	2.28 (2.08–2.49)	<0.001	
Low birth weight (n = 747)#					
<18.5	1.45 (0.89–2.38)	2.06 (1.47–2.88)	0.97 (0.56–1.67)	0.052	1.73 (1.35–2.22)
18.5–23.9	1.17 (0.86–1.61)	1.00	0.77 (0.60–0.99)	0.022	1.00
24.0–27.9	1.21 (0.31–4.67)	1.38 (0.85–2.24)	0.76 (0.57–1.02)	0.047	0.92 (0.72–1.16)
≥28.0	3.87 (0.45–33.33)	1.35 (0.34–5.42)	0.85 (0.59–1.24)	0.144	0.96 (0.68–1.35)
P for trend	0.647	<0.001	0.841		<0.001
Total	1.05 (0.81–1.37)	1.00	0.67 (0.55–0.81)	<0.001	

* Adjusted for maternal age, maternal height, maternal education, smoking, family income, and maternal occupation.

# Adjusted for maternal age, maternal height, maternal education, smoking, family income, maternal occupation, and gestational age.

## Discussion

The present study indicated that maternal prepregnancy obesity and excessive GWG were associated with greater risks of pregnancy-induced hypertension, caesarean delivery, and greater infant size at birth. Meanwhile, maternal prepregnancy underweight was associated with increased risks of infant SGA and low birth weight, and maternal inadequate GWG was associated with increased risks of infant preterm delivery and SGA.

Several studies found that the risk of pregnancy-induced hypertension was greater among women who entered prepregnancy with overweight or obesity, and who had excessive GWG [3], [22]–[24]. The Avon Longitudinal Study of Parents and Children (ALSPAC) found that greater GWG in early pregnancy (up to 18 weeks) was independently associated with an increased risk of gestational hypertension, and GWG in midpregnancy (18–29 weeks) was not associated with blood pressure change in late pregnancy (29–36 weeks) [24]. Obesity is known as one important risk factor for pregnancy related hypertension and preeclampsia [25]. Frederick *et al.* found that every 1 kg/m^2^ increase in prepregnancy BMI resulted in an 8% increased risk of preeclampsia (adjusted RR = 1.08; CI = 1.05–1.11) [26]. Obese women have been shown to have increased blood volume and cardiac output, and increased blood pressure during pregnancy [8], [27]. Thus, women who develop hypertension during pregnancy are more likely to experience edema than women who remain normotensive, and this in turn may result in greater GWG. In the present study, women who were prepregnancy obese and had excessive GWG showed an almost 6-fold risk of pregnancy-induced hypertension compared with women with normal prepregnancy BMI and adequate GWG. In addition, we also found that women with prepregnancy overweight or obesity and adequate GWG had a higher risk of pregnancy-induced hypertension. Our findings indicate that higher prepregnancy BMI might play an important role in the development of pregnancy-induced hypertension.

In the present study, the relative risks of GDM were higher in those women with prepregnancy overweight and obesity. In the joint 12 analyses of maternal prepregnancy BMI and GWG, women with prepregnancy overweight or obesity and adequate GWG had a 2.6–3.6 fold risk of GDM, and women with prepregnancy overweight or obesity and excessive GWG had a 1.6–2.2 fold risk of GDM compared with those women with normal weight and adequate GWG. Thus, our findings indicate that higher prepregnancy BMI plays an important role in the development of GDM. Previous studies reported that GDM was an adverse pregnancy outcome of excessive GWG [28], [29]. However, like some other studies [30], the present study did not find an association of excessive GWG with GDM risk. This might be that women who were diagnosed as GDM would take more lifestyle interventions and control weight gain during pregnancy. In addition, a previous study showed insulin sensitivity might increase or decrease during early pregnancy depending on the prepregnancy insulin sensitivity status of the women. In the very insulin-sensitive women, insulin sensitivity most often decreases and is accompanied by an increase in adipose tissue [31]. In contrast, among more insulin-resistant women (e.g. those have GDM), insulin sensitivity often increases and is accompanied by a decrease in potential loss of adipose tissue [32]. These physiologic changes may help to explain in part the relative no more weight gain during pregnancy in GDM women.

The positive associations of maternal higher prepregnancy BMI and excessive GWG with the risk of larger birth weight of infants were similar to previous studies [3], [11], [33]. A clear association exists between maternal obesity and infant size at birth. In recent years researchers have recognized that excessive GWG is also associated with increased weight at birth [3]. In the present study, maternal excessive GWG had a 2.32 fold risk of infant LGA compared with those women with adequate GWG. Similarly, mothers with prepregnancy overweight or obesity had a 1.73–2.80 fold risk of infant LGA compared with those mothers with normal prepregnancy weight. A recent study reported that the greatest difference in neonatal fat mass was observed among prepregnancy overweight women with excessive GWG compared with overweight women with adequate GWG [34]. For women within the excessive GWG category, infants born to normal weight mothers had lower percent body fat (11.8%) than infants born to overweight mothers (13.7%) and obese mothers (14.2%). Infants born to mothers with excessive GWG had greater fat-free mass than infants born to mothers with adequate GWG [34]. This indicated that maternal excessive GWG might play an important role as prepregnancy BMI in the offspring's overweight, and might contribute to the overweight epidemic among infants and children. One important issue of reverse causality should be also considered in the analyses of maternal GWG with infant LGA. It has been suggested that associations of maternal GWG with infant LGA do not result from GWG itself, but rather to underlying factors that influence both weight gain and the outcomes, such as maternal diet composition and physical activity level. In addition, it is important to determine whether these relationships are independent of prepregnancy BMI or if they differ by prepregnancy BMI. The present study indicated that the positive association of maternal GWG and the risk of infant LGA was consistent among women different prepregnancy BMI and independent of maternal prepregnancy BMI.

In the present study, we also found that maternal higher prepregnancy BMI and excessive GWG were associated with caesarean delivery. This may be that large size baby birth could cause delivery complications, such as caesarean delivery. A US study reported that the rate of caesarean delivery was 27.2% in women who gained more than the weight that the IOM recommended [9]. Another study reported that increased prepregnancy BMI was associated with an increasing incidence of caesarean section in a population of Chinese women in Hong Kong [35]. The rate of caesarean delivery in the present study (65.6%) was higher than in other studies from developed areas, but similar to a previous study in urban areas of China (64.1%) [36]. The higher rate of caesarean section in China may be influenced by socioeconomic factors such as education, household income, and access to health insurance. The introduction of the one-child policy in 1979 may have contributed indirectly to the rise. Parents who expect to have only one child may prefer birth by caesarean section to vaginal delivery because they think it is safer and free from pain and anxiety.

The present study evaluated the single and joint associations of maternal prepregnancy BMI and GWG with maternal and neonatal outcomes. We found that maternal prepregnancy BMI plays a more important role than GWG in maternal outcomes, especially in pregnancy complications. Pregnancy-induced hypertension and gestational diabetes are the two key common pregnancy complications. Previous studies have reported that maternal obesity is associated with increased risks of adverse pregnancy outcomes including gestational diabetes and pregnancy-induced hypertension [3], [37]. Women with prepregnancy overweight or obesity would take more lifestyle interventions and control weight gain during pregnancy, and these two diseases will both affect weight gain in pregnancy. However, the present study found that only women with prepregnancy underweight and adequate GWG had decreased risks of pregnancy-induced hypertension and caesarean section compared with women with normal prepregnancy weight and adequate GWG. And maternal prepregnancy underweight with excessive GWG was associated with an increased risk of caesarean section. So, it is important to help women gain adequate weight during pregnancy based on their prepregnancy BMI to improve pregnancy outcomes. For neonatal outcomes, both higher prepregnancy BMI and excessive GWG could result in high maternal glucose, free fatty acid, and amino acid concentrations, thus leading to the risk of greater infant size at birth. Therefore, maternal prepregnancy BMI has similar effects as GWG in the neonatal outcomes.

The major strength of our study is the use of GWG category instead of net weight gain according to the new IOM guidelines [8]. These new guidelines are formulated as a range of weight gain for each category of prepregnancy BMI. Our study assessed the single and joint associations of maternal prepregnancy BMI and GWG with the risk of pregnancy and neonatal outcomes. A limitation of our study is that women in the present study are all living in urban areas. We did not include information of women who live in rural areas. However, the present study is an ongoing project, and we will obtain more information from both urban and rural areas. Another limitation is that the numbers of part of pregnancy outcomes in several multiple cells are low in the joint analyses of maternal prepregnancy BMI and GWG with pregnancy outcomes, which may limit statistical power in some subgroups.

In summary, our study indicated that pregnancy-induced hypertension, caesarean delivery, and infant size at birth were important outcomes of maternal prepregnancy overweight/obesity and excessive GWG. Health care providers should inform women to enter pregnancy with a BMI in the normal weight category and limit their GWG to the range specified for their prepregnancy BMI. It is important to pay more attention to maternal influences during pregnancy to prevent the intergenerational cycle of obesity. Strategies to raise public awareness of the risks of maternal adiposity and weight gain during pregnancy on offspring's future health are required.

## Supporting Information

Table S1 Numbers of subjects of maternal and neonatal outcomes by joint effects of maternal prepregnancy body mass index and weight gain during pregnancy.
(DOC)
