# Supplementary material for: Maternal Prepregnancy Body Mass Index and Gestational Weight Gain on Pregnancy Outcomes
Source: PLoS One. 2013 Dec 20;8(12):e82310. doi: 10.1371/journal.pone.0082310 (PMC3869661; doi:10.1371/journal.pone.0082310)
Supplement: Table S1 — Numbers of subjects of maternal and neonatal outcomes by joint effects of maternal prepregnancy body mass index and weight gain during pregnancy. (DOC) [file pone.0082310.s001.doc]

Table S1. Numbers of subjects of maternal and neonatal outcomes by joint effects of maternal prepregnancy body mass index and weight gain during pregnancy

| Prepregnancy body mass index (kg/m^2^) | Institute of Medicine categories | | | Total |
| --- | --- | --- | --- | --- |
|  | Inadequate | Adequate | Excessive |  |
| Numbers of subjects |  |  |  |  |
| <18.5 | 735 | 1 845 | 1 229 | 3 809 |
| 18.5-23.9 | 2 494 | 8 534 | 10 914 | 21 942 |
| 24.0-27.9 | 87 | 749 | 5 349 | 6 185 |
| ≥28.0 | 24 | 99 | 1 914 | 2 037 |
| Total | 3 340 | 11 227 | 19 406 | 33 973 |
| Numbers of gestational diabetes (n = 1721) |  |  |  |  |
| <18.5 | 17 | 56 | 18 | 91 |
| 18.5-23.9 | 157 | 418 | 362 | 937 |
| 24.0-27.9 | 8 | 89 | 397 | 494 |
| ≥28.0 | 1 | 15 | 183 | 199 |
| Total | 183 | 578 | 960 | 1 721 |
| Numbers of pregnancy-induced hypertension (n = 742) |  |  |  |  |
| <18.5 | 4 | 11 | 12 | 27 |
| 18.5-23.9 | 41 | 112 | 186 | 339 |
| 24.0-27.9 | 1 | 29 | 173 | 203 |
| ≥28.0 | 2 | 10 | 161 | 173 |
| Total | 48 | 162 | 532 | 742 |
| Numbers of caesarean section (n = 22 295) |  |  |  |  |
| <18.5 | 378 | 968 | 754 | 2 100 |
| 18.5-23.9 | 1 470 | 5 068 | 7 300 | 13 838 |
| 24.0-27.9 | 59 | 537 | 4 059 | 4 655 |
| ≥28.0 | 18 | 85 | 1 599 | 1 702 |
| Total | 1 925 | 6 658 | 13 712 | 22 295 |
| Numbers of preterm delivery (n = 1050) |  |  |  |  |
| <18.5 | 32 | 46 | 22 | 100 |
| 18.5-23.9 | 127 | 291 | 222 | 640 |
| 24.0-27.9 | 5 | 42 | 163 | 210 |
| ≥28.0 | 2 | 3 | 95 | 100 |
| Total | 166 | 382 | 502 | 1 050 |
| Numbers of large for gestational age (n = 3 544) |  |  |  |  |
| <18.5 | 15 | 55 | 84 | 154 |
| 18.5-23.9 | 126 | 553 | 1 328 | 2 007 |
| 24.0-27.9 | 7 | 71 | 845 | 923 |
| ≥28.0 | 5 | 20 | 435 | 460 |
| Total | 153 | 699 | 2 692 | 3 544 |
| Numbers of small for gestational age (n = 3 111) |  |  |  |  |
| <18.5 | 149 | 324 | 117 | 590 |
| 18.5-23.9 | 359 | 870 | 749 | 1 978 |
| 24.0-27.9 | 6 | 67 | 354 | 427 |
| ≥28.0 | 3 | 10 | 103 | 116 |
| Total | 517 | 1 271 | 1 323 | 3 111 |
| Numbers of macrosomia (n = 3 318) |  |  |  |  |
| <18.5 | 14 | 55 | 83 | 152 |
| 18.5-23.9 | 117 | 512 | 1 257 | 1 886 |
| 24.0-27.9 | 5 | 72 | 796 | 873 |
| ≥28.0 | 4 | 17 | 386 | 407 |
| Total | 140 | 656 | 2 522 | 3 318 |
| Numbers of low birth weight (n = 746) |  |  |  |  |
| <18.5 | 26 | 65 | 18 | 109 |
| 18.5-23.9 | 94 | 199 | 146 | 439 |
| 24.0-27.9 | 4 | 31 | 104 | 139 |
| ≥28.0 | 2 | 3 | 54 | 59 |
| Total | 126 | 298 | 322 | 746 |
